# Cadherin-6 type 2, K-cadherin (CDH6) is regulated by mutant p53 in the fallopian tube but is not expressed in the ovarian surface

**DOI:** 10.18632/oncotarget.11499

**Published:** 2016-08-22

**Authors:** Subbulakshmi Karthikeyan, Daniel D. Lantvit, Dam Hee Chae, Joanna E. Burdette

**Affiliations:** ^1^ Center for Biomolecular Sciences, Department of Medicinal Chemistry and Pharmacognosy, College of Pharmacy, University of Illinois at Chicago, Chicago, IL 60607, USA

**Keywords:** high-grade serous ovarian carcinoma, p53 mutation, fallopian tube, tissue specific mutant p53 transgenic mouse model, cadherins

## Abstract

High-grade serous ovarian cancer (HGSOC) is the most lethal gynecological malignancy and may arise in either the fallopian tube epithelium (FTE) or ovarian surface epithelium (OSE). A mutation in p53 is reported in 96% of HGSOC, most frequently at R273 and R248. The goal of this study was to identify specific gene targets in the FTE that are altered by mutant p53, but not in the OSE. Gene analysis revealed that both R273 and R248 mutant p53 reduces CDH6 expression in the oviduct, but CDH6 was not detected in murine OSE cells. p53^R273H^ induced SLUG and FOXM1 while p53^R248W^ did not induce SLUG and only modestly increased FOXM1, which correlated with less migration as compared to p53^R273H^. An oviduct specific PAX8^Cre/+^/p53^R270H/+^ mouse model was created and confirmed that *in vivo* mutant p53 repressed CDH6 but was not sufficient to stabilize p53 expression alone. Overexpression of mutant p53 in the p53 null OVCAR5 cells decreased CDH6 levels indicating this was a gain-of-function. SLUG knockdown in murine oviductal cells with p53^R273H^ restored CDH6 repression and a ChIP analysis revealed direct binding of mutant p53 on the CDH6 promoter. NSC59984, a small molecule that degrades mutant p53^R273H^, rescued CDH6 expression. In summary, CDH6 is expressed in the oviduct, but not the ovary, and is repressed by mutant p53. CDH6 expression with further validations may aide in establishing markers that inform upon the cell of origin of high grade serous tumors.

## INTRODUCTION

Ovarian cancer is the fifth leading cause of cancer related deaths in American women [1]. The American Cancer Society projects 22,280 new cases of ovarian cancer in 2016 and the estimated 5 year death rate is 65% [1]. Epithelial ovarian cancer (EOC) constitutes the most predominant form of the disease with high grade serous ovarian cancer (HGSOC) being the most common and lethal histotype [2]. One of the obstacles in developing treatments for HGSOC is the lack of understanding of the pathogenesis of HGSOC due to an uncertain site of origin [3]. Traditionally, it was thought that HGSOC arises from the ovarian surface epithelium (OSE), but over the last decade it has become apparent that the fallopian tube epithelium (FTE) is also a likely source for HGSOC [4]. As evidence, prophylactically removed fallopian tubes from women who are genetically predisposed to developing ovarian cancer expressed dysplastic and hyperplastic lesions with accumulation of mutations in the tumor suppressor gene psmall proteoglycan associated with53 in the FTE [5]. Marquezet et al. and Merritt et al. found significant correlations between the transcriptome of serous ovarian cancers and normal fallopian tube epithelium and a decrease in overall survival for fallopian tube like tumors [6, 7]. A retrospective study found that salpingectomy was associated with a 45% reduction in the risk of ovarian cancer [8]. Lastly, a mouse model with *Pax8* driven tissue specific *Brca* and *Pten* deletion combined with *Tp53* mutation in fallopian tube secretory epithelium leads to HGSOC [9].

Mutations in p53 occur in 96% of HGSOC, and the identification of p53 mutations in putative benign lesions suggests that mutation of this gene is critically important and occurs early in fallopian-tube derived HGSOC [10]. In HGSOC, the most frequent p53 mutations occur in the DNA binding domain at codons R273, R248 and R175 [11]. Some DNA binding mutations are termed gain of function (GOF) mutations, which refers to the enhanced biological activity that facilitates tumor growth and metastasis [12]. Mouse models with p53^R270H/−^ and p53^R172H^ develop more carcinomas, with increased capacity for metastasis [13]. Mice expressing a knock-in p53^R248W^ have accelerated formation of lymphomas and sarcomas with increased chemoresistance [14]. Ovarian carcinoma patients harboring a R248W mutation have a poor overall survival compared to R273H with selective chemoresistance to microtubule stabilizers [15]. Currently, small molecules are being identified that can alter the mutant p53 configuration back to wild-type or can degrade mutant p53 protein. Treatment with NSC59984 in p53^R273H^ mutant colorectal cancer cell lines demonstrated an increase in mutant p53 degradation and stabilization of p53^WT^ signaling through activation of p73 [16]. Given the evidence that mutation in p53 impacts tissues differently, the response of HGSOC derived from OSE and fallopian tube to small molecules that alter mutant p53 may differ.

Murine oviductal epithelial cells (MOE), the equivalent of human fallopian tube, harboring the p53^R273H^ mutation migrate more than control cells [17]. Microarray data confirmed expression changes of pro-migratory genes in p53^R273H^ transfected MOE cells compared to parental cells [17]. The tissue specificity of the pro-migratory genes remains unknown. However, the same mutation did not show any phenotypic changes in murine ovarian surface epithelial cells (MOSE) [17] largely due to a lack of SLUG induction. SLUG is a p53 transcriptional target and a migratory protein [18]. Different p53 mutations have tissue specific signaling mechanisms in other cancers. For example, a study in pediatric adrenal cortical carcinoma found that p53^R337H^ did not form sarcoma in soft tissues or bone which is frequently found in case of Li-Fraumeni families [19]. Another study found tissue specific regulation of p53 targets in liver and spleen cells [20]. Liver cells had induced p21 induction with no expression of apoptotic genes, but spleen cells had the inverse, specific induction of apoptotic gene PUMA occurred without changes in p21 [20].

The objective of this study was to identify a mutant p53 FTE target gene to determine if markers might be present that can facilitate determination of the cell of origin. Two frequently reported p53 DNA contact mutants (R273H and R248W) in HGSOC were chosen and a panel of pro-migratory genes from our previously published cDNA microarray data in FTE vs OSE was mined to determine if they are differentially regulated by mutant p53 in the OSE compared to oviductal cells. In addition, NSC59984 treatment in MOE cells harboring p53^R273H^ and p53^R248W^ suggests that p53 DNA binding mutants may differ and may require different small molecules to inhibit their activity.

## RESULTS

### CDH6 is decreased by p53 mutation in MOE cells not MOSE cells

**Figure 1:**
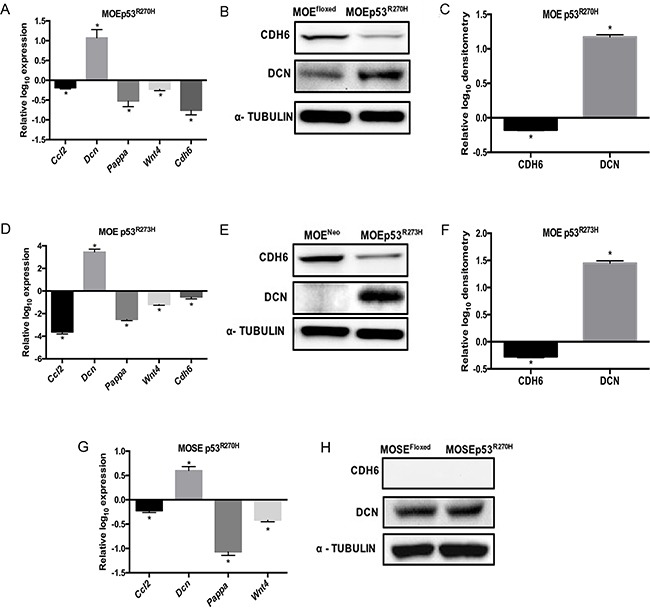
Comparative pro-migratory gene expression levels in MOE and MOSE cells Pro-migratory mRNA levels were measured by qPCR in MOE and MOSE cells with p53 mutation and normalized to their respective controls. **A.** MOEp53^R270H^ relative to MOE^Floxed^ control cells. Cell lysates were probed with CDH6 and DCN antibodies. α - Tubulin is used as a loading control. **B.** Western blot on MOE^Floxed^ and MOEp53^R270H^ cells. **C.** Densitometry analysis of MOEp53^R270H^ relative to MOE^Floxed^ control cells. **D.** qPCR data obtained from MOEp53^R273H^ relative to MOE^Neo^ control cells. **E.** Western blot on MOE^Neo^ and MOEp53^R273H^ cells. **F.** Densitometry analysis of MOEp53^R273H^ relative to MOE^Neo^ control cells. **G.** qPCR data obtained from MOSEp53^R270H^ relative to MOSE^Floxed^ control cells. **H.** Western blot on MOSE^Floxed^ and MOSEp53^R270H^ cells. Data represent mean ± SEM. Student *t*-test was used to determine significance, (*p < 0.05) relative to control.

A microarray analysis in MOE cells expressing the p53^R273H^ revealed a significantly altered pro-migratory gene signature compared to the MOE vector control cells [17]. Based on those results, several candidate genes (Supplementary Table S1) were chosen based on their expression in the fallopian tube, their role in migration, and their association with cancer [21–25]. The selected candidate genes were cadherin-6 type 2, K-cadherin (*Cdh6,* cell adhesion proteoglycan), pregnancy-associated plasma protein A (*Pappa,* secreted metalloprotease), wingless-type MMTV integration site family, member 4 (*Wnt4,* local signaling molecule) and decorin (*Dcn,* small proteoglycan associated with collagen). Quantitative PCR (qPCR) was used to validate the microarray using MOE cells expressing p53^R270H^ mutation (the murine equivalent of R273H). *Ccl2* expression was repressed by mutant p53 [26] and this was confirmed as a positive control (Figure 1A). Increased *Dcn,* decreased *Pappa*, *Cdh6* and *Wnt4* expression levels were measured in MOE p53^R270H^ (Figure 1A) cells compared to control. Next, CDH6 and DCN protein levels were examined. Reduced CDH6 and increased DCN protein was seen in MOE cells with p53^R270H^ (Figure 1B and 1C).

To test if human p53 mutations (R273H and R248W) alter the pro-migratory gene expression in MOE cells, qPCR was performed. MOE cells harboring human p53 mutation R273H exhibited induced *Dcn* expression and reduced *Cdh6*, *Pappa*, *Wnt4* and *Ccl2 mRNA* levels compared to control cells (Figure 1D). Western blot analysis confirmed that p53^R273H^ repressed CDH6 expression and induced DCN expression in MOE cells (Figure 1E and 1F) similar to the murine p53^R270H^. To further determine if any of these targets are uniquely regulated in fallopian tube cells compared to the OSE, MOSE cells expressing p53^R270H^ mutation (the murine equivalent of R273H) were used. qPCR revealed increased *Dcn* and reduced *Ccl2*, *Pappa* and *Wnt4* in MOSEp53^R270H^ cells compared to control cells (Figure 1G). Interestingly, *Cdh6* expression was not detected in MOSE cells. Consistent with qPCR analysis, CDH6 protein was not detected and DCN expression was not altered in MOSE cells with p53^R270H^ (Figure 1H). These analyses revealed that CDH6 is repressed in MOE cells by p53 mutation, but its expression and regulation in MOSE cells was not detectable.

**Figure 2:**
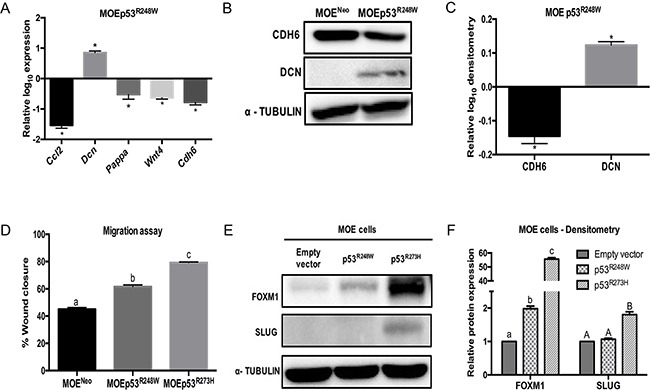
p53^R248W^ reduces CDH6 expression and increases migration in MOE cells **A.** qPCR analysis on MOEp53^R248W^ cells relative to MOE^Neo^ control cells. Cell lysates were probed with CDH6 and DCN antibodies. α - Tubulin is used as a loading control. **B.** Western blot on MOE^Neo^ and MOEp53^R248W^ cells. **C.** Densitometry analysis of MOEp53^R248W^ relative to MOE^Neo^ control cells. **D.** Migration assay of MOE^Neo^, MOEp53^R273H^, and MOEp53^R248W^ cells 8 hours after wounding. **E.** FOXM1 and SLUG western blot. α - Tubulin is used as loading control. **F.** Densitometry analysis on relative FOXM1 and SLUG protein levels compared to empty vector cells. Data represent mean ± SEM. Data was analyzed with a student *t*-test (*p < 0.05 relative to control) or one-way ANOVA (a–c or A-B bar without common letter differ, *p* < 0.05).

MOE cells with stable p53^R248W^ expression were next investigated. A MOEp53^R248W^ clone was confirmed for human p53 mRNA (Supplementary Figure S1A) and p53 protein expression (Supplementary Figure S1B and S1C). p53^R248W^ increased *Dcn* and reduced *Ccl2*, *Cdh6*, *Pappa* and *Wnt4* as compared to control (Figure 2A). CDH6 protein levels were decreased and DCN was induced due to p53^R248W^ in MOE cells (Figure 2B and 2C) similar to p53^R273H^. Because p53^R273H^ and p53^R248W^ are the two most frequently mutated sites in ovarian cancer, and our previous data indicated that p53^R273H^ mutation enhanced migration [17], we tested the effect of stable p53^R248W^ on migration. MOEp53^R248W^ cells migrated ~20% faster than the vector control (MOE^Neo^ cells), but not as rapidly as MOE p53^R273H^ cells (Figure 2D). FOXM1 is induced by mutant p53 and can increase tumor metastases [27]. Our previous data indicated that SLUG was a key downstream target of R273H that mediated motility [27]. FOXM1 expression levels in MOE p53^R248W^ cells were lower than the levels in MOE p53^R273H^ cells (Figure 2E & 2F). SLUG expression was not detected in MOE p53^R248W^ cells (Figure 2E & 2F). These data suggest that p53^R273H^ and p53^R248W^ do not equally induce SLUG and FOXM1 expression in the fallopian tube and this modified migration.

### The PAX8^cre/+^p53^R270H/+^ mouse model confirms CDH6 is regulated in the oviducts and not the ovaries

**Figure 3:**
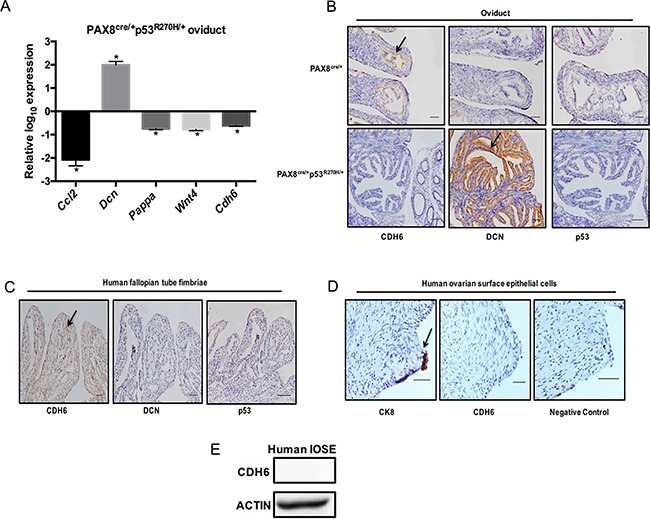
CDH6 is repressed by mutant p53 in murine oviducts **A.** qPCR data on the pro-migratory genes mRNA levels from PAX8^cre/+^p53^R270H/+^ oviducts relative to Pax8^cre/+^ oviducts. **B.** Immunohistochemistry analysis of CDH6, DCN and p53 staining in Pax8^cre/+^ and PAX8^cre/+^p53^R270H/+^ oviducts **C.** Immunohistochemistry on human fallopian tube for CDH6, DCN and p53. Black arrow indicates positive staining. Scale bars = 100 μm. **D.** Immunohistochemistry on human ovaries for CDH6 and CK8. Black arrow indicates positive staining. Scale bars = 20 μm. **E.** CDH6 western blot in normal human IOSE cells. Actin is used as loading control. Data represent mean ± SEM. Student *t*-test was used to determine significance, (*p < 0.05) relative to control.

In order to verify whether CDH6 is regulated by mutant p53 *in vivo*, a transgenic mouse model was developed. A tissue specific transgenic mouse model was generated by crossing mice with a Lox-stop-Lox site regulating expression of the R270H mutation with mice expressing cre-recombinase driven by the *Pax8* promoter. Using this model, p53^R270H^ remains floxed and only p53^WT^ is expressed except in PAX8 expressing tissues, such as the oviduct, uterus, and kidney. PCR confirmed that the p53^R270H^ had recombined in the oviduct and uterus, but not in the ovaries, which do not express PAX8 and therefore would lack cre-recombinase (Supplementary Figure S2A). Mice were sacrificed after 9 months, tissues were dissected and mRNA was extracted for qPCR. *Cdh6, Pappa, Wnt4,* and *Ccl2* mRNA levels were decreased and *Dcn* mRNA was increased in PAX8^cre/+^p53^R270H/+^ compared to control PAX8^cre/+^ oviducts (Figure 3A). *Dcn, Pappa, Wnt4,* and *Ccl2* were not significantly altered in the PAX8^cre/+^p53^R270H/+^ ovaries (Supplementary Figure S2B). Consistent with the cellular models, *Cdh6* mRNA levels were not detected in ovaries. Immunohistochemistry (IHC) analysis was used to identify the protein levels in PAX8^cre/+^p53^R270H/+^ and PAX8^cre/+^ oviducts and ovaries. CDH6 staining was high in PAX8^cre/+^ oviducts and the staining intensity decreased in PAX8^cre/+^p53^R270H/+^ oviducts as predicted based on repression from p53^R270H^ (Figure 3B). DCN was detected in PAX8^cre/+^ and was induced in PAX8^cre/+^p53^R270H/+^ oviducts (Figure 3B). p53 stabilization was not detected in PAX8^cre/+^p53^R270H/+^ oviducts (Figure 3B). Negative CDH6 and p53 staining was seen in the ovaries (Supplementary Figure S2C). Both PAX8^cre/+^p53^R270H/+^ and PAX8^cre/+^ ovaries stained positive for DCN with no change at the protein level by R270H (Supplementary Figure S2C). Normal human fallopian tube fimbriae also expressed CDH6 (Figure 3C). DCN expression and p53 stabilization was not observed in normal human fallopian tube fimbriae (Figure 3C). CDH6 staining was not detected in normal human ovarian surface epithelium by IHC (Figure 3D). CK8 positive staining confirmed the presence of OSE (Figure 3D). Additionally, CDH6 protein was not detected in immortalized ovarian surface epithelial cell lysate (IOSE80) (Figure 3E). These *in vivo* analyses demonstrate that CDH6 is specifically repressed by mutant p53 in the oviducts.

### Mutant p53 reduces CDH6 expression independently and through SLUG induction in HGSOC

**Figure 4:**
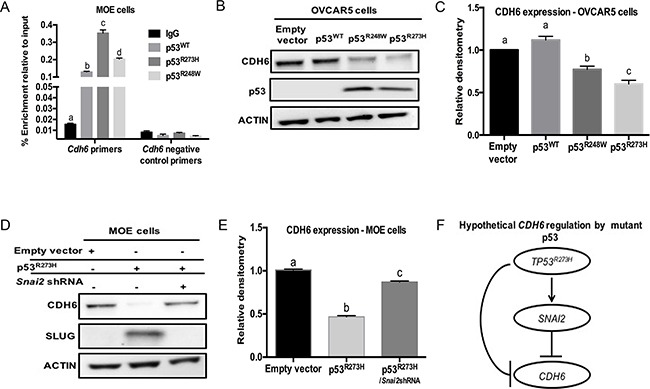
Mutant p53 repress CDH6 independent of p53^WT^ in human HGSOC cell lines **A.** ChIP analysis on MOE cells for non-specific IgG, p53^WT^, p53^R273H^ and p53^R248W^ occupancy on *Cdh6* promoter. Primers designed on non - p53-binding site used as negative control primers. **B.** OVCAR5 cells were transiently transfected with empty vector, p53^WT^, p53^R273H^ and p53^R248W^. Western blot analysis for CDH6 and p53 levels. Actin is used as loading control. **C.** Densitometry analysis for CDH6 expression levels on transiently transfected OVCAR5 cells relative to actin. **D.** Western blot image for CDH6 and SLUG levels in MOE cells. **E.** Densitometry data obtained on CDH6 expression levels in MOE cells with empty vector, p53^R273H^ and p53^R273H/*Snai2*shRNA^ stable expression. **F.** Hypothetical pathway for CDH6 regulation by p53^R273H^ in HGSOC. Data represent mean ± SEM. One-way ANOVA was used to determine, a – d (*p* < 0.05) bars without common letter differ.

Mutations in p53 may directly or indirectly repress *Cdh6* promoter activity. To determine if mutant p53 regulates CDH6 through direct transcriptional repression, MOE cells harboring p53^WT^, p53^R273H^, and P53^R248W^ were cultured and ChIP analysis was performed using p53 and Non–specific IgG antibodies. *Mdm2* and *Atf* were used as positive controls in ChIP analysis (Supplementary Figure S3A and S3B) [28]. Increased p53^WT^ occupancy was observed on the *Cdh6* promoter when compared to control IgG (Figure 4A). The p53^R273H^ and p53^R248W^ demonstrated a significantly higher occupancy on the *Cdh6* promoter compared to p53^WT^ (Figure 4A). These findings suggest that p53 mutation decreases *Cdh6* expression in oviductal epithelium through direct repression of the promoter.

A panel of human HGSOC cell lines including OVCAR3, OVCAR5, OVKATE, OVSAHO and OVCAR8 were tested for the presence of CDH6. CDH6 protein was detected in OVCAR5, OVKATE, OVSAHO and OVCAR8 cells (Supplementary Figure S3C). A weak expression of CDH6 was observed in OVCAR3 cells (Supplementary Figure S3C). In order to decipher the regulation of CDH6 by mutant p53 in the absence of p53^WT^, a p53^null^ human HGSOC cell line (OVCAR5 cells) was transfected to express p53^WT^, p53^R273H^ or p53^R248W^. ****Endogenous CDH6 protein expression did not change in p53^WT^ compared to empty vector control transfected cells (Figure 4B & 4C). CDH6 expression was decreased by p53^R273H^ and p53^R248W^ in OVCAR5 cells (Figure 4B & 4C). These results suggest that CDH6 was repressed by mutant p53 and that its repression was not dependent on blocking p53^WT^ protein.

To identify if SLUG, a mutant p53 induced pro-migratory protein can reduce CDH6, MOE cells with stable p53^R273H^ expression and *Snai2* knock down were used. MOEp53^R273H^ cells had increased SLUG expression and stable SLUG knock down significantly decreased cell migration compared to control cells [17]. SLUG can repress cadherin expression by binding to the E-box motifs on their promoters [29]. Knockdown of SLUG restored CDH6 protein compared to MOEp53^R273H^ cells (Figure 4D & 4E). Therefore, both SLUG and mutant p53 regulate CDH6 (Figure 4F).

### NSC59984 rescues CDH6 expression and inhibits cell migration in MOE cells

**Figure 5:**
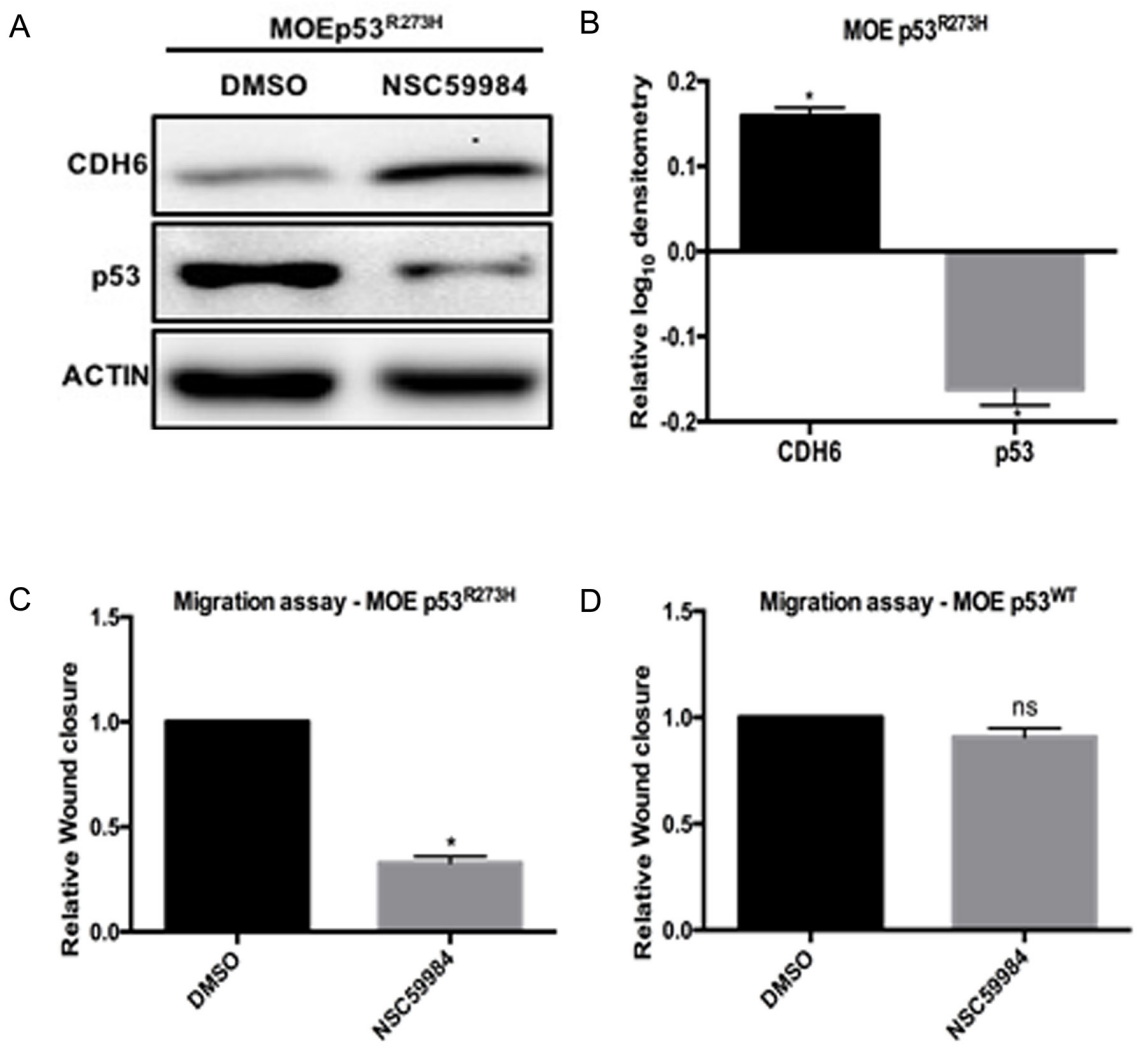
NSC59984 degrades p53^R273H^, restores CDH6 and inhibits cell migration in MOE cells **A.** MOE cells withp53^R273H^ and p53^R248W^ cells were treated with 25 μM/L NSC59984 for 8 hours. Cells lysates were probed for CDH6 and p53. Western blot image is represented and Actin is used as loading control. **B.** Densitometry analysis on CDH6 and p53 expression levels with NSC59984 treatment in MOEp53^R273H^ cells relative to DMSO treated cells. **C.** Migration assay after 8 hours of scratch in MOEp53^R273H^ cells with 25 μM/L NSC59984. **D.** Migration assay after 8 hours of scratch in MOEp53^WT^ cells with 25 μM/L NSC59984. Data represent mean ± SEM. Student *t*-test was used to determine significance, (*p < 0.05) relative to control. n.s – not significant.

In order to study if inhibition of mutant p53 activity can rescue CDH6 repression, MOE cells with p53^R273H^ were treated with NSC59984. NSC59984 is a small molecule that stabilizes wild type p53 signaling and increases the degradation of mutant p53 [16]. NSC59984 degraded mutant p53 protein and rescued CDH6 repression in MOE cells with p53^R273H^ (Figure 5A and 5B). NSC59984 also reduced the cell migration in MOEp53^R273H^ with no change in migration in MOEp53^WT^ cells compared to DMSO treated cells (Figure 5C and 5D). HGSOC cell line OVCAR3 was selected to test the activity of NSC59984 because it expresses the p53^R248W^ mutation and is considered a viable model of high grade serous cancer [30]. NSC59984 did not have an effect on OVCAR3 cells (Supplementary Figure S4). Our results demonstrate that NSC59984 reduces p53^R273H^ activity, but not R248W, and can revive CDH6 repression and reduces cell migration.

## DISCUSSION

Mounting evidence suggests that the FTE is a likely progenitor cell for HGSOC with p53 mutation almost being essential in HGSOC [5–7, 10]. However, the ovarian surface may still give rise to some serous tumors [31]. While almost all tumors have a p53 mutation, the two most frequent mutations are the DNA binding missense mutations, R273H and R248W [12, 32]. One potential route to improving personalized therapy for ovarian cancer is to understand the accumulating steps in tumor formation that might be targeted, which could be specific to the tissue from where the tumors originate. Thus if a tissue specific target of mutant p53 exists, it may help to differentiate FTE from OSE derived tumors. Using previously published microarray data on MOEp53^R273H^ cells [17] a set of pro-migratory genes including *Cdh6, Pappa, Dcn, Wnt4* and *Ccl2* were chosen to study in oviductal epithelium and OSE. CDH6 was repressed by p53 mutants only in the MOE cells, but it was not expressed or regulated in MOSE cells. Human fallopian tube epithelial cells express CDH6, but CDH6 expression was not detected in human OSE. While gene expression for mutant p53 targets may not be retained throughout all phases of tumor progression, these data suggest that unique mutant p53 targets are present in fallopian tube that may provide clues to distinguish the two cell progenitor populations. This study also identified that p53 mutations are capable of altering a subset of genes identically in fallopian tube and ovarian surface. CDH6 is membrane glycoprotein and a member of the cadherin family that mediates homophilic cell-to-cell adhesion. CDH6 plays a key role in cell morphogenesis and when disrupted, may contribute to cell migration [33, 34]. CDH6 has been associated with cancer cells in the literature as a target that is repressed by estrogen signaling in ovarian cancer [21]. Cristofaro et al. demonstrated PAX8 can directly bind to CDH6 promoter and induce its expression in an immortalized fallopian tube secretory epithelial cell line [35]. Lastly, CDH6 was identified as a downstream target for p53 and Pax2 co-operative regulation during kidney development and nephrogenesis [36].

MOE cells stably transfected with p53^R248W^ migrated more than control cells, but this was lower than p53^R273H^ cells, consistent with previous reports suggesting that R273H increases tumor invasion [12, 32]. R273H and R248W may have distinct regulation on certain pro-migratory targets, for example SLUG and FOXM1 were only highly regulated by R273H. However, this study cannot fully explain the longer survival times in patients with R273 compared to R248 [32], which may be a reflection of the chemoresistance often seen in tumors with R248 [12, 32].

In HGSOC, p53 signatures (p53 protein stabilization in FTE) are a proposed early precursor lesions [37]. Intriguingly, Pax8^cre/+^p53^R270H/+^ oviducts lacked p53 staining by IHC, which is consistent with published data that mutation in p53 alone did not result in p53 stabilization in murine models [13, 17]. In other models with constitutive mutant p53 expression, the stabilization of the protein is seen in tumors, but not in the adjacent normal cells with a p53 mutation [38]. Terzian et al. found mice lacking *Mdm2* or *p16^Ink4a^* stabilized mutant p53 when crossed with p53 homozygous mutant mice suggesting that loss of heterozygosity, loss of MDM2, or loss of p16 are necessary to mediate p53 stabilization [39]. The Pax8^cre/+^p53^R270H/+^ animals demonstrated no signs of tumor formation up to 12 months whereas our previous results using stable cells lines derived from oviductal cells with stable PTEN knockdown alone demonstrated p53 stabilization [40]. These data suggest that mutation alone may not generate the p53 signature, but may be a prerequisite for p53 stabilization.

High mutant p53 occupancy compared to the p53^WT^ on the *Cdh6* promoter was demonstrated by ChIP analysis in MOE cells. SLUG knockdown restored CDH6 repression in MOE cells with p53^R273H^ mutation. SLUG, a p53 transcriptional target, was enhanced by p53^R273H^ and increased migration [17]. SLUG can bind to E-box motifs to repress cadherin expression [41]. The TCGA reports amplification of *SNAI2* (encodes for SLUG) in 12% HGSOC. Together, the data suggests that p53 independently and coupled with SLUG can regulate CDH6 expression. CDH6 levels were significantly reduced when p53^R273H^ and p53^R248W^ were expressed even in the absence of wild-type p53. This supports that CDH6 repression by mutant p53 is independent of p53^WT^ and may be due to GOF activity. Zhu et al. found that p53 GOF mutants, including R273 and R248, in a CHIPseq analyses had proximal peaks on chromatin regulatory genes in breast cancer cell lines [42]. The same study also found that p53^R273H^ had enriched peaks on E26 transformation-specific (ETS) motifs that were distinct from p53^WT^ [42]. The CDH6 promoter contains an ETS binding motif. This observation support that CDH6 repression by mutant p53 could be due to GOF activity and this is consistent with mechanisms in the literature that require ETS motifs [42].

Currently, researchers are trying to identify small molecules to increase the degradation of mutant p53 or revert the mutant confirmation to wild type. A cell-penetrating peptide significantly inhibited p53 aggregation in OVCAR3 cells resulting in reduced tumors *in vivo* [43]. NSC59984 is a small molecule that can effectively degrade mutant p53 protein via MDM2 mediated ubiquitination, and is effective against colorectal cancer [16]. NSC59984 enhanced CDH6 expression, reduced mutant p53 expression, and inhibited cell migration in MOEp53^R273H^ cells. NSC59984 did not have an effect on p53 or CDH6 protein in OVCAR3 cells harboring the p53^R248W^ mutation. These data indicate that p53 mutants are unique and may require distinct small molecules to inhibit their activity, however more exploration is required to support this finding. Additionally, future clinical studies using small molecules or peptides that inhibit mutant p53 aggregation or that degrade mutant p53 should therefore be considered along with sequencing HGSOC tumors prior and post treatment. The existence of a p53 DNA contact mutant target, which is regulated in the fallopian tube but not expressed in ovaries, with further validations using human ovarian tumors and HGSOC clinical samples, may add to the existing tools for finding the cell of origin of serous tumors and improve personalized therapies that work better in tumors arising from the fallopian tube.

## MATERIALS AND METHODS

### Cell culture

Murine oviductal epithelial (MOE) cells were obtained from Dr. Barbara Vanderhyden at the University of Ottawa and were maintained in media as previously described [17]. MOE^Neo^, MOE^floxed^, MOEp53^R273H^, MOEp53^R270H^, MOSE^floxed^ and MOSEp53^R270H^ cell lines were made as previously described [17]. MOEp53^R248W^ stable cell lines were generated using a construct pCMV-Neo-BAM p53 R248W which was a gift from Bert Vogelstein (Addgene plasmid # 16437) [44]. Stable clones were selected using Neomycin resistance and were verified with Western blot and qPCR analysis. OVCAR3 cells were obtained from ATCC and maintained in media as described previously [45]. Small molecule NSC59984 was available through National Cancer Institute (NCI) as part of NCI/DTP Open Chemicals Repository. OVCAR5 cells (gift from Dr. Gustavo Rodriguez and Dr. Teresa Woodruff at Northwestern University) and are available through NCI as part of the NCI60 tumor cell line anticancer drug screen and maintained in media as described previously [46]. Human Immortalized ovarian surface epithelial cells (IOSE80) were a gift from Dr. Nelly Auersperg at the University of Vancouver and were maintained as described previously [46]. OVKATE and OVSAHO were obtained through MTA from the Japanese Collection of Research Bioresources Cell Bank (JCRB) and maintained in media as described previously [47]. OVCAR8 cells were obtained from ATCC and maintained in media as described previously [47]. OVCAR3, OVCAR5, OVKATE, OVSAHO and OVCAR8 cells have been verified by STR analysis. The molecular profiles and *in vivo* tumor growth capabilities of the human HGSOC cell lines used in this study have been previously characterized [47]. OVCAR5 cells were transiently transfected with antibiotic resistant plasmids containing gene of interests which includes pCMV6-Myc-Neo (Origene, donated by Dr. Kwong Wong, M.D. Anderson Cancer Center, Houston, TX), pCMV-Neo-BAM p53 R273H was a gift from Bert Vogelstein (Addgene plasmid # 16439) [44], pCMV-Neo-BAM p53 wt was a gift from Bert Vogelstein (Addgene plasmid # 16434) [44] and pCMV-Neo-BAM p53 R248W. All transfections were performed using TransIT LT1^TM^ (Mirus Bio, Madison, WI) according to the manufacturer's instructions.

### Animals

All animals were treated in accordance with the National Institutes of Health Guidelines for the Care and Use of Laboratory Animals and the established Institutional Animal Use and Care protocol at the University of Illinois at Chicago (UIC). In addition, the Animal Care Committee approved the protocol 14-163. Animals were housed in a temperature and light controlled environment (12 hours' light, 12 hours dark) and were provided food and water *ad libitum*. The Lox-stop-Lox regulating p53^R270H^ mice (from Mouse models of Human Cancer Consortium) were bred with mice that express Cre- Recombinase (from Research institute of molecular pathology, Vienna, Dr. Bohr-Gasse [48] under the control of *Pax8* promoter to generate p53^R270H/+^ mice. Genotyping was done as previously described (Jackson Laboratory, Bar Harbor, ME) to identify p53^R270H/+^ mice from p53^Cre/+^ mice. All mice were euthanized by CO_2_ inhalation followed by cervical dislocation. Reproductive tract was extracted and used for immunohistochemistry and qPCR analysis.

### Immunohistochemistry (IHC)

Reproductive tract was prepared for paraffin sectioning and immunohistochemistry or hematoxylin and eosin stain as described previously [49]. Tissues were incubated with the following primary antibodies at 4°C overnight: CDH6 1:50 (cat No: ab197845, Abcam, Cambridge, MA), decorin (DCN) 1:50 (Cat No: PA5-13538, ThermoFisher scientific, Rockford, IL), Cytokeratin 8 (CK8) 1:100 (Developmental studies, Hybridoma Bank, Iowa city, IA) and p53 1:50 (Cat No: SC6243, Santa Cruz Biotechnology, Dallas, TX). In all experiments, tissues without the primary antibody treatment were used as a negative control. Images were acquired on a Nikon Eclipse E600 microscope using a DS-Ri1 digital camera and NIS Elements software (Nikon Instruments).

### Quantitative reverse transcriptase PCR (qPCR) and standard PCR

RNA extraction was performed using Trizol (Life Technologies, Grand Island, NY) and chloroform with isopropanol precipitation followed by ethanol washes and DNAse step. RNA concentrations were determined using NanoVue plus spectrophotometer (GE healthcare, product code 28-9569-62). 1μg of RNA was reverse transcribed using iScript cDNA synthesis kit (Biorad, Hercules, CA) according to manufacturer's instructions. All qPCR measurements were performed using the ABI ViiA7 (Life Technologies, San Diego, CA) and SYBR green (Roche, Madison, WI). All primers were validated for efficiency through serial dilutions and generation of a standard curve and visual inspection of the melt curve. Standard PCR was done on the mouse models for genotyping and to demonstrate cre-mediated expression of the mutant p53 allele in PAX8-cre expressing tissues. The primers and the protocol are as described previously [9]. Primers used in qPCR are listed in Supplementary Table S2.

### Western blot analyses

Cells were lysed using RIPA buffer (50mM Tris, pH 7.6, 150 mM NaCl, 1% Triton X-100, 0.1% SDS) with protease and phosphatase inhibitors (Sigma-Aldrich, St. Louis, MO). Protein concentration was determined by BCA assay (Pierce, Rockford, IL). 30 μg of total protein was electrophoresed in 10% SDS – PAGE gel and transferred to nitrocellulose membrane (Thermo Fisher Scientific, Waltham, MA). Blots were then blocked with 5% milk in TBS-T or 5% BSA in TBS-T and probed at 4°C overnight with primary antibodies. The following primary antibodies were used: ACTIN 1:1000 (Cat No: A2066, Sigma-Aldrich, St. Louis, MO), CDH6 1:500 (cat No: ab197845, Abcam, Cambridge, MA), DCN 1:1000 (Cat No: PA5-13538, ThermoFisher scientific, Rockford, IL), p53 1:500 (Cat No: SC6243, Santa Cruz Biotechnology, Dallas, TX), FOXM1 1:200 (Cat No: SC500, Santa Cruz Biotechnology, Dallas, TX), SLUG 1:1000 (Cat No: ab106077, Abcam, Cambridge, MA and α – Tubulin 1: 1000 (Cat No: 2144, Cell Signaling, Beverly, MA). Anti-rabbit HRP-linked secondary antibodies (Cell Signaling Technology, Inc.) were used at a concentration of 1:1000 for all blots except for actin, which was used at 1:10,000 (Promega, Madison, WI) for 30 min in blocking buffer. After washing, membranes were incubated in SuperSignal West Femto substrate (Thermo Scientific, Rockford, IL) before imaging on a FlourChem™ E system (ProteinSimple, Santa Clara, CA). Densitometric analysis was performed using NIH ImageJ.

### Migration assay

Cells were plated to confluence (1.5 x 10^5^ cells/well) in a 24- well plate. A uniform wound was created through the cell monolayer. Cells were washed with 1X phosphate buffer saline (PBS) and replenished with new media. Pictures were taken at 0 and 8 hrs after scratching using an AmScope MU900 with Toupview software (AmScope, Irvine, CA). The area of the scratch was analyzed with ImageJ NIH software. Percentage of closure was determined by measuring the final volume of the wound relative to the initial volume of the scratch.

### Chromatin immunoprecipitation (ChIP analysis)

30 μl Dynabeads protein G (Life technologies, Cat no. 10003D) were used per pull down assay. Bead washing and antibody binding was performed as described previously [50]. A magnetic separation rack (Cell signaling – 7017) was used to pull the beads off between washes. Normal rabbit IgG 1:100 (Cat No: 2729, Cell signaling, Beverly, MA) was used as control and p53 1:100 (Cat No: SC6243, Santa Cruz Biotechnology, Dallas, TX) as test antibody. MOEp53^WT^ and MOEp53^R273H^ and MOEp53^R248W^ cells in 10cm dish were fixed with 1% formaldehyde. MOEp53^WT^ were treated with 10 μM proteasome inhibitor MG132 (Sigma - M7449) for 4 hours prior to fixing. Cell lysis and cross-linking was done as described previously [50]. Sonication was done with 20 second pulse “on”, 40 second pulse “off” for a total of 4 minutes, with 50% amplitude using a Sonic Dismembrator (Branson, Model 500). Cross-linking was achieved by mixing 250 μl sonicated supernatant and 100 μl of Protein G bead slurry attached to IgG and p53 antibodies separately. De-crosslinking and dissociation of chromatin-antibody complexes from the beads was done as described previously [50]. Phenol chloroform extraction was performed using UltraPure™ Phenol: Chloroform: Isoamyl Alcohol (25:24:1 v/v) as described by manufacturer's protocol (Thermo Fisher Scientific, Waltham, MA). Combining 3.6 μl dH_2_O, 0.5 μM primer pair, 5 μl SYBR green mix and 1 μl sample qPCR analysis was done using the ABI ViiA7 (Life Technologies, San Diego, CA). The relative occupancy of the immunoprecipitated protein (IgG and p53) on the target gene promoter was estimated using the following equation: 2ˆ(Mean Ct ^Input-log100^_2_ – Mean Ct^IgG or^ ^p53^). Primers used are listed in Supplementary Table S3.

### Statistical analyses

Data are presented as the mean ± standard error of the mean. Statistical analysis was carried out using GraphPad Prism software (GraphPad, La Jolla, CA). Statistical significance was determined by Student's t-test or one-way ANOVA followed by a Tukey's posthoc test. p<0.05 considered significant.

## SUPPLEMENTARY FIGURES AND TABLES


