# Supplementary material for: Cadherin-6 type 2, K-cadherin (CDH6) is regulated by mutant p53 in the fallopian tube but is not expressed in the ovarian surface
Source: Oncotarget. 2016 Aug 22;7(43):69871–82. doi: 10.18632/oncotarget.11499 (PMC5342521; doi:10.18632/oncotarget.11499)
Supplement: Supplementary file 1 [file oncotarget-07-69871-s001.pdf]

Cadherin-6 type 2, K-cadherin (CDH6) is regulated by mutant p53 in the fallopian tube but is not expressed in the ovarian surface

SUPPLEMENTARY FIGURES AND TABLES

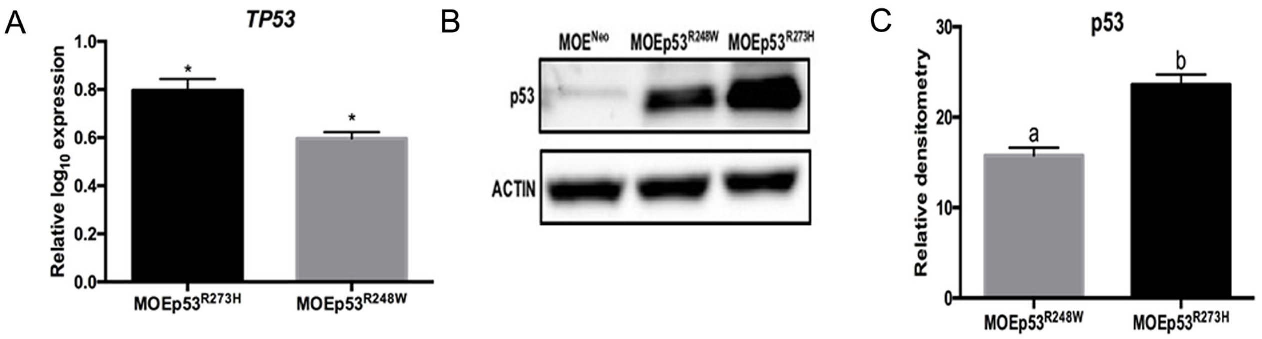

Supplementary Figure S1: p53 mRNA and protein levels in MOEp53^{R248W} cells to validate the stable transfections. **A.** qPCR showing p53 mRNA levels in MOE cells stably transfected with p53^{R248W} relative to MOE^{Neo} control cells. MOEp53^{R273H} cells were used as a positive control. **B.** p53 western blot image. **C.** Densitometry analysis on p53 levels in MOE cells with p53^{R273H} and p53^{R248W}. Data represent mean ± SEM. Student *t*-test was used to determine significance, (*$p < 0.05$) relative to control. One-way ANOVA was used to determine, a – b ($p < 0.05$) bars without common letter differ.

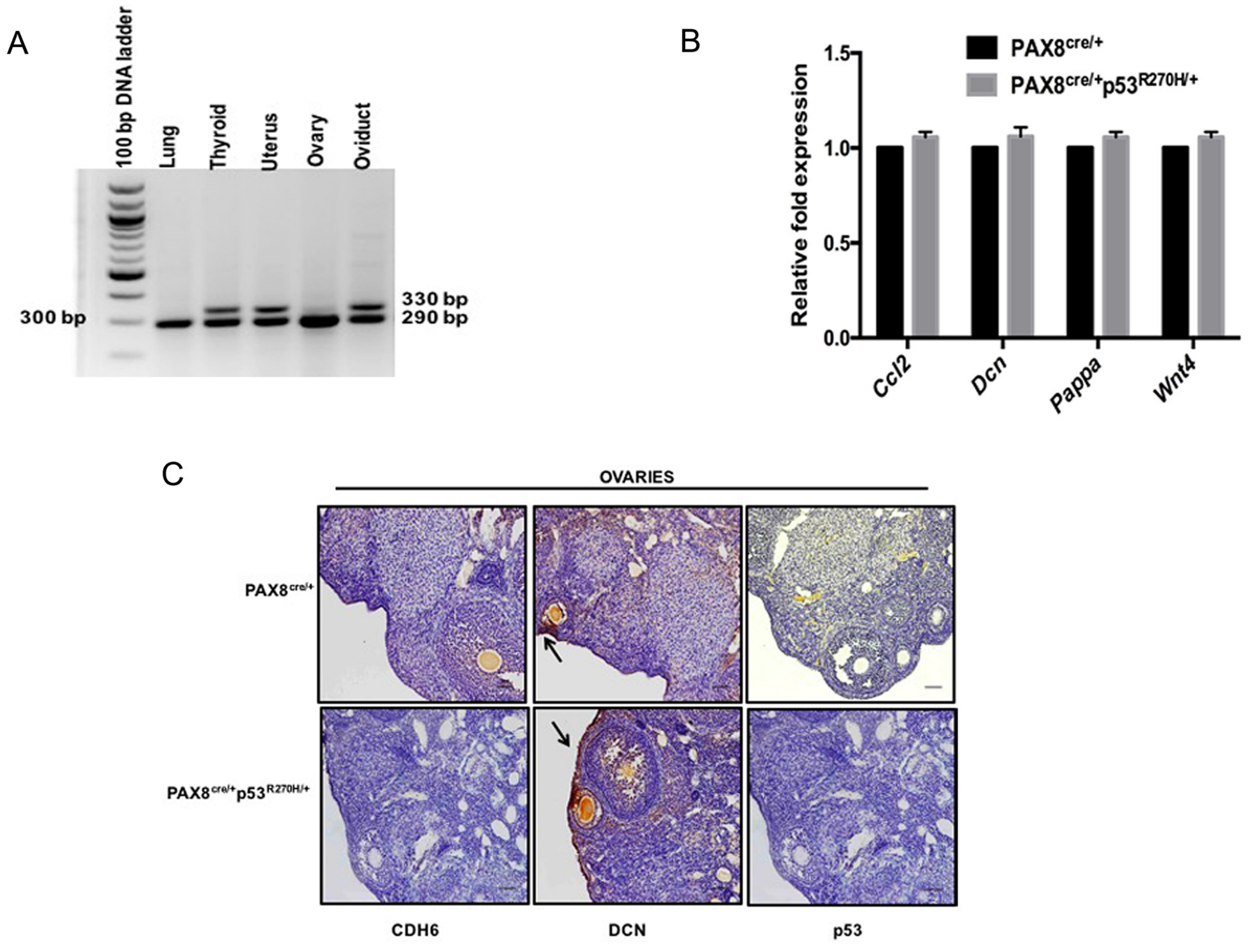

Supplementary Figure S2: CDH6 is not detected in MOSE cells and murine ovaries. **A.** Standard PCR was performed on the genomic DNA extracted from tissues of PAX8$^{cre/+}$p53$^{R270H/+}$ mice using primers specific to *loxP* sites. Agarose gel image showing recombined mutant p53 band at 330 bp in PAX8 expressing tissues and p53^{WT} positive band at 290 bp in all tissues tested. **B.** qPCR showing no significant change in pro-migratory gene expression in PAX8$^{cre/+}$p53$^{R270H/+}$ ovaries compared to Pax8$^{cre/+}$ ovaries. **C.** Immunohistochemistry analysis on Pax8$^{cre/+}$ and PAX8$^{cre/+}$p53$^{R270H/+}$ ovaries with absence of CDH6 and no change in DCN expression. p53 staining is used as negative control. Black arrow indicates positive staining. Data represent mean ± SEM. Scale bars = 50 µm.

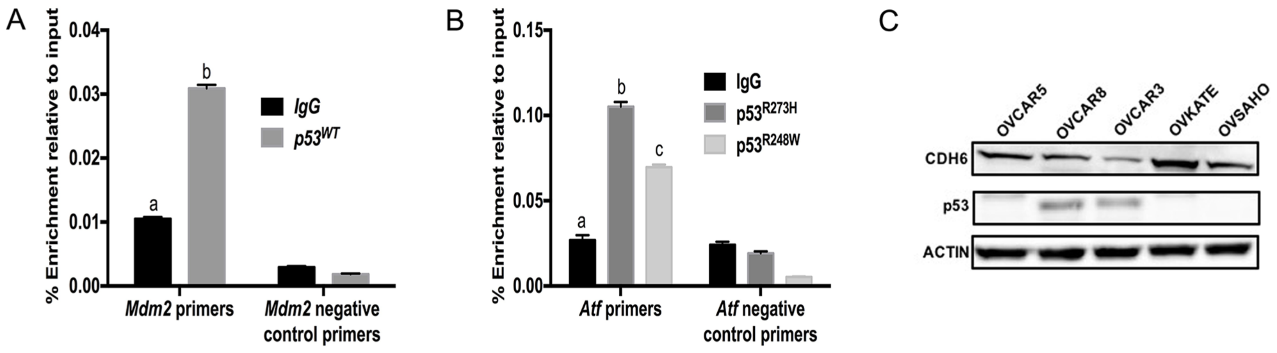

Supplementary Figure S3: ChIP analysis positive controls and CDH6 expression in human HGSOC cell lines. *Mdm2* and *Atf* are used as positive controls for $p53^{WT}$ and mutant p53 occupancy respectively. **A.** ChIP analysis showing increased $p53^{WT}$ occupancy on *Mdm2* promoter compared to IgG. **B.** ChIP analysis showing high $p53^{R273H}$ and $p53^{R248W}$ occupancy on *Atf* promoter compared to IgG. Primers designed on non-p53-binding site in the promoters is used as controls. **C.** CDH6 and p53 western blot in human HGSOC cell lines. Data represent mean ± SEM. One-way ANOVA was used to determine, a – c ($p < 0.05$) bars without common letter differ.

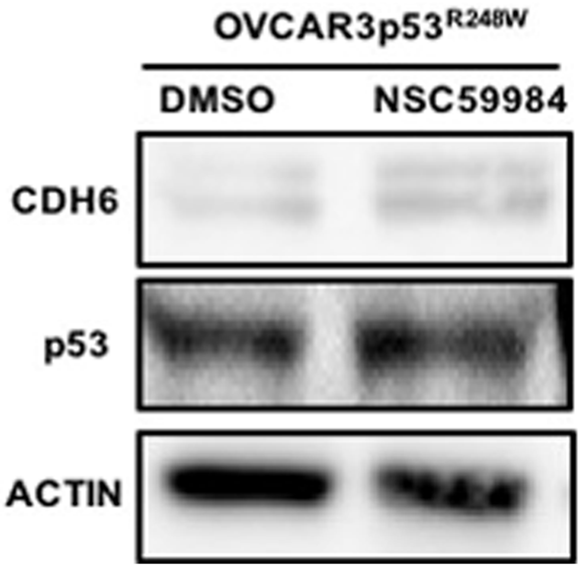

Supplementary Figure S4: NSC59984 did not degrade $p53^{R248W}$ in OVCAR3 cells. CDH6 and p53western blot in OVCAR3$p53^{R248W}$ cells treated with 25 µM/L NSC59984 for 8 hours. Actin is used as loading control.

Supplementary Table S1: Table showing the list of genes chosen for the study and fold change from microarray

Genes	Log_{10} Fold change	P value
Ccl2	-1.50314	< 0.0001
Dcn	1.4153	< 0.0001
Cdh6	-0.62727	< 0.0001
Pappa	-0.2883	< 0.01
Wnt4	-0.9896	< 0.0001

Supplementary Table S2: Table showing the list of primer sequences used for qPCR

Gene	Forward primer (5'-3')	Reverse primer (5'-3')
Ccl2	GTCCCTGTCATGCTTCTGG	GCTCTCCAGCCTACTCATTG
Dcn	CTGGCCAATGTTCCTCATC	GGTAGACGACCTGGATATACTT
Pappa	CTTTGCCTAGAAGGGAGAATC	ACATCTGGGTGACCTTCT
Wnt4	CAGGAAGGCCATCTTGAC	ACCGTCAAACTTCTCCTTTAG
Cdh6	GATCCGATTATCAGTACGTGGG	TGTATGTCGCCTGTGTTCTC

Supplementary Table S3: List of primers used in ChIP analysis

Gene	Forward primer (5'-3')	Reverse primer (5'-3')
Mdm2	GTCCCTGTCATGCTTCTGG	GCTCTCCAGCCTACTCATTG
Atf	GCAGGTAAACCCCAGGCGT	GGCAGGTAGGAGGAGATCCCA
Cdh6	GCAAGTGAATGAAGTGGGAGGGA	CTGTCAAGGGGAGGGGGC
Mdm2 negative control	GTGCACCGTGTGCAAACAGTG	GATAAGGTGGCTCAGCCCTCTTG
Atf negative control	GCTTGATCCGATTCCTTCGCTTC	CAGCCTCACTCGCTGTTGTGA
Cdh6 negative control	GAGAGGGTGAGACACAGTTGTTCT	CATGGAGAGGGCAAAGGGTACA
